# Study on the Scale-Up Possibility of a Combined Wet Grinding Technique Intended for Oral Administration of Meloxicam Nanosuspension

**DOI:** 10.3390/pharmaceutics16121512

**Published:** 2024-11-25

**Authors:** Csilla Bartos, Anett Motzwickler-Németh, Dávid Kovács, Katalin Burián, Rita Ambrus

**Affiliations:** 1 Institute of Pharmaceutical Technology and Regulatory Affairs, Faculty of Pharmacy, University of Szeged, 6720 Szeged, Hungary; bartos.csilla@szte.hu (C.B.); nemeth.anett@szte.hu (A.M.-N.); kovacsdav97@gmail.com (D.K.); 2 Department of Medical Microbiology, Albert Szent-Györgyi Medical School, University of Szeged, 6720 Szeged, Hungary; burian.katalin@med.u-szeged.hu

**Keywords:** combined wet grinding/milling, scale-up method, meloxicam, nanonization, dissolution, cytotoxicity

## Abstract

**Background/Objectives:** This article reports on the scalability of a combined wet grinding technique applying planetary ball mill and ZrO_2_ pearls as the grinding medium. After the determination of the parameters in a laboratory scale, the tenfold scale-up method was set. Meloxicam (MEL) was used as a nonsteroidal anti-inflammatory drug (NSAID) intended for per os delivery. During grinding, the PVA solution was used as a dispersion medium. **Methods:** The influence of the scaling-up on the particle size, morphology, crystallinity, and intra- and interparticulate phenomena has been studied. Formulation investigations of the milled suspensions were carried out. The dissolution test and the cytotoxicity analyses were accomplished. **Results:** Submicron MEL particle-containing samples were produced in both grinding scales. After the particle size determination was achieved from the suspensions, the wet milled, dried products were studied. The particle size of the dried products fell into the same range for both scales of milling (the maximum particle size was about 580 nm). There was no significant difference in drug crystallinity after the grindings; 70% of MEL remained crystalline in both cases. A remarkable interaction between the components did not develop as a result of milling. The polarity of the products increased, which resulted in a better dissolution, especially in the case of intestinal fluid (~100% in the first 5 min). The products were not found to be toxic. **Conclusions:** This research demonstrates that the scaling-up of combined wet grinding technique is feasible by adjusting the milling parameters and the adequate amount of excipient.

## 1. Introduction

Nearly 40–60% of approved active pharmaceutical ingredients—which are most often administered through the oral route—are poorly water soluble. Solving this problem is a perennial challenge for the pharmaceutical industry [1,2]. Particle engineering techniques are generally applied to modify the physico-chemical and biopharmaceutical properties of drugs [3]. The various size reduction techniques include top-down methods, where the raw material is subsequently broken down until micro- or nanosized particles are produced [4]. The reduction in particle size of these drugs may change their crystalline structure, increasing their dissolution rate and absorption, and therefore ensuring better bioavailability, decreased therapeutic dose, and more a favorable side effect profile [5]. There is an overview of dry and wet grinding techniques (ball milling, media milling, high pressure homogenization, etc.) in the literature which discusses the grinding-induced amorphization and as a result the enhanced dissolution in the case of many drugs (furosemide, indomethacin, phenytoin, ibuprofen, glibenclamide, etc.) [6]. In the wet grinding procedure, a concentrated dispersion of drug particles in an aqueous or organic solvent liquid medium is performed. For wet grinding, additives are needed, independently of the preparation of micro- or nanoparticles. The choice of stabilizer is specific for each drug candidate and each formulation procedure. Stabilizers help minimize the aggregation of suspended particles via electrostatic and steric mechanisms [7]. In addition, the applied additives are usually hydrophilic in nature (such as polyvinylpyrrolidone, cellulose ethers, polyethylene glycol, polyvinyl alcohol, poloxamers, and cyclodextrins) and ensure hydrophilicity to the hydrophobic drug particle surfaces, which therefore results in better wettability and dissolution properties [8,9,10]. Applying wet milling, the preparation of suspensions is possible, which can be feasible for the development pharmaceutical formulations by means of drying or by direct processing [11].

Grinding is the most commonly used surface machining process, which is carried out with the aim of obtaining a result with a specific shape in the chosen material both in a laboratory scale and in the industry, because of its reproducibility, and the process is relatively easy to implement [12,13,14]. During the development of pharmaceutical formulations, lab-scale milling is followed by the scale-up of the process, which poses a challenge for the industry due to its complexity [15]. There is some research on the investigation of the scale-up of grinding based on mathematical models. Mannheim determined the power consumption of the stirred ball mill for scale-up by a method based on dimensional analysis [16]. Lehocký et al. have studied transferring the stirred media wet milling process from a batch to a flow-through arrangement during process scale-up. The parametric dependence of the particle breakage kinetics on the main process parameters has been systematically investigated. They were ascertained that the same terminal particle size could be obtained by several different combinations of the ball fill level, stirring rate, and overall process time [17]. Lestary et al. investigated the scalability of wet ball milling. He investigated factors including milling time, milling speed, and the type of mill. They determined that during the change in the milling mode from batch mode to recirculation mode, either the milling speed or milling duration needed to be adjusted to ensure that enough energy was given to break the particles into a smaller size [18]. The guidelines or a published method for the scaling-up of planetary ball mill are not yet known [19]. Grinding effects depend on different milling conditions such as the pot diameter, ball diameter, ball-filling ratio, revolution radius, and rotational speed, etc. First, the critical parameters of the lab-scale grinding need to be determined. Mio et al. have studied a computational simulation based on the Discrete Element Method for the scale-up of a planetary ball mill [20]. They established that the impact energy is a key factor in controlling the milling achievement; this indicates the guideline for the designing relationships of the directions and the speed ratio between the rotation of the pot and the revolution of the disk in a planetary ball mill. During the investigation of the scale-up method by using the impact energy of balls, it was determined that the milling rate increased with an increasing rotation speed and the specific impact energy, and it also increased with decreasing the sample weight. Using computational simulation, the relationship between the impact energy and the total scale-up ratio was defined. The impact energy of the balls in the planetary mill is proportional to the diameter and depth of the pot and the revolution radius [21].

Meloxicam (MEL) is an enolic acid that belongs to the oxicam class of NSAIDs that selectively inhibits COX-2 (cyclo-oxygenase-2). It is usually used in the treatment of pain or inflammation [22]. MEL has a crystalline structure, low water-solubility (4.4 μg/mL at 25 °C), and a low dissolution rate. Different particle size reduction techniques were previously applied in the literature in order to change the dissolution features of MEL. Co-grinding of MEL with PVP-C30 in a planetary ball mill resulted in a significant increase in the dissolution properties [23]. Formulation of the MEL nanosuspension by an emulsion–diffusion method also resulted in a significantly faster dissolution compared to the untreated particles [24]. The preparation of MEL nanocrystals by wet-milling technology and high-pressure homogenization improved its solubility, dissolution, and oral bioavailability [25]. The formulation of smartcrystals, applying a bead mill method, followed by high-pressure homogenization resulted in the efficient dissolution of the drug as well [26].

For nanoparticles, intended for utilization in therapeutic tools to treat human diseases, cytotoxicity studies are required to investigate their effects on the contacted cells. The cytotoxicity of nanoparticles depends on the experimental conditions and their physico-chemical properties [27]. You et al. determined that in case of MEL nanocrystals, the cell viability decreased in time- and dose-dependent manners; furthermore, the cytotoxicity increased with the increasing particle size [25]. MEL micro- and nanoparticles, produced by nanosecond laser ablation, showed a concentration-dependent cytotoxicity when different laser wavelengths were used [28]. Extra-fine particles containing nanosized meloxicam, prepared by nano spray-drying, revealed no cytotoxic effect in the studied concentration range [29].

To eliminate the weakness of the conventional grinding techniques, our research group has developed and optimized a new combined method for particle size reduction in MEL, combining planetary ball and pearl milling [30]. Due to the high efficiency of smaller beads sizes [31] and the high mechanical energy of the planetary mill [32], the particle size reduction to the nano range was implemented. For the nanonization of MEL, poly(vinyl alcohol) (PVA), as a stabilizing additive, was required, which promotes the milling efficiency. The process parameters of combined milling (pearl amount, milling time, and rotation speed) were optimized in a laboratory scale and the influence on the particle size distribution, crystallinity, and dissolution rate of MEL was also studied. The changes in polymer viscosity and the drug–polymer interaction were also determined [33]. For these investigations, a small milling chamber was applied, which had a volume of 50 mL. However, the following question arose: could the process be scaled-up using a ten-times larger chamber?

This article reports on a comparison of a laboratory scale and a 10-fold scaled-up combined wet grinding process, as a novel organic solvent-free technology, where the planetary ball mill was combined with the pearl grinding procedure using a small (50 mL) and a larger (500 mL) milling chamber. Our aim was to scale-up the nanonization of MEL in an aqueous dispersant medium, intended for oral administration. The effects of both millings on the particle size, physical-chemical features, resuspendability, holding time, drug content, and uniformity were analyzed. The in vitro dissolution of MEL was studied and evaluated after filling the suspensions into capsules. The cytotoxicity study of products was also performed. In our opinion, this work presents useful information, filling a gap between the laboratory and industrial scale production of nanoparticle-based formulations.

## 2. Materials and Methods

### 2.1. Materials

MEL (4-hydroxy-2-methyl-N-(5-methyl-2-thiazolyl)-2H-benzothiazine-3-carboxamide-1,1-dioxide) was attained from EGIS Ltd. (Budapest, Hungary). PVA (polyvinyl alcohol), was procured from Sigma Aldrich (St. Louis, MO, USA). Purified water was used to prepare the 5% PVA solution. The phosphate buffer (pH 7.4 ± 0.1) was made from Na_2_HPO_4_·2H_2_O (Spektrum 3D, Debrecen, Hungary), the pH was adjusted with 85% o-phosphoric acid (Molar Chemicals Kft, Halásztelek, Hungary). The artificial gastric fluid (pH 1.2 ± 0.1) was made from NaCl (Molar Chemicals Kft., Halásztelek, Hungary) and 37% HCl (Merck Kft., Budapest, Hungary) while the intestinal fluid (pH 6.8 ± 0.1) was made from KH_2_PO_4_ (Molar Chemicals Kft, Halásztelek, Hungary) and NaOH (Spektrum 3D, Debrecen, Hungary). All these chemical reagents were of analytical grade.

### 2.2. Preparation of Suspensions

**Figure 1:**
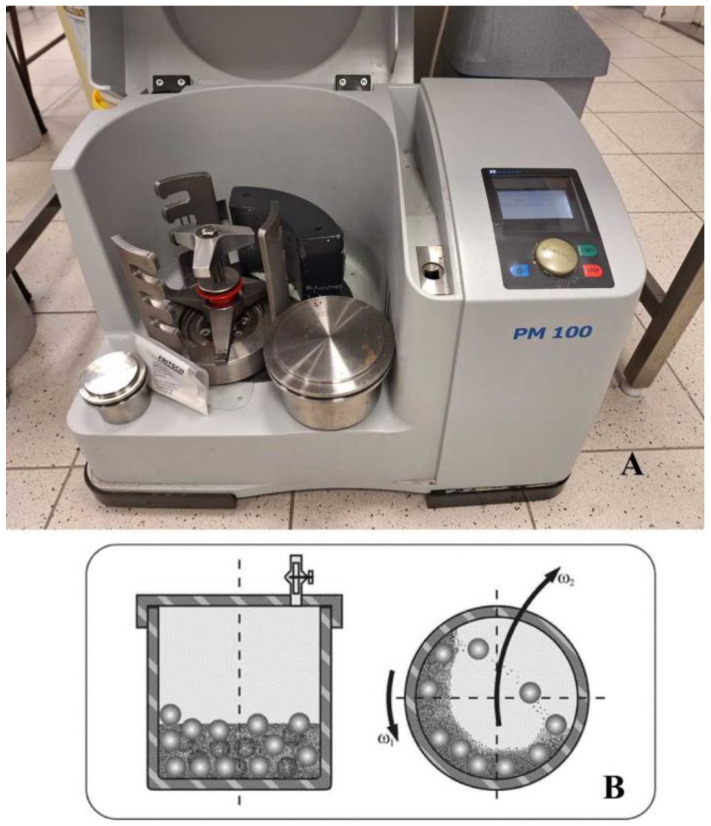
The picture of the planetary ball mill (**A**) and the schematic view of the ball mill (**B**).

For investigating the possibility of scale-up in the case of combined wet grinding (a combination of planetary ball and bead grinding) (Figure 1), the suspension intended for per os application was prepared using the large grinding chamber (500 mL) of the planetary ball mill (Retsch PM 100) (Retsch GmbH, Haan, Germany). As a grinding medium, 0.3 mm ZrO_2_ beads were applied. A total of 20 g of MEL was first suspended in the 5% PVA solution (180 g). Rpm—required for scale-up—was calculated from rpm, determined for lab-scale milling (Ω_1_^2^d_M1_ = Ω_2_^2^d_M2_, where Ω represents the revolutions per minite, and d_M_ the diameter of the chamber (mm). (Ω_1_ = 500 rpm, d_M1_ = 4.5 cm, d_M2_ = 10 cm)) [21,29]. The calculated prm was 335. During the grinding procedure, samples were taken at times of 10, 30, 50, 70, 90, 110, 130, and 150 min in order to monitor the particle size changes. Pearls were removed using a 150 μm mesh size sieve. The sample milled in the large milling chamber (MELscup) was compared with the sample prepared in a small volume chamber (500 rpm, 50 min, 50 mL chamber) (MELlab). As references, the initial drug (rawMEL) and the suspension of untreated MEL in the PVA solution (MELsusp) were applied. The abbreviations used in the article are summarized in Table 1.

### 2.3. Determination of Particle Size Distribution

After grinding, the particle size of MEL in the suspensions was established. The volume-based particle size distribution (PSD) was determined by laser diffraction (Mastersizer 2000, Malvern Instruments Ltd., Worcestershire, UK) with the following parameters: 300RF lens; small volume dispersion unit (2500 rpm); refractive index for dispersed particles 1.720; refractive index for dispersion medium 1.330. The Dynamic Laser Light Scattering method was applied to determine the PSD. The particle size of MEL was determined immediately from the ground suspension. The size analysis was repeated three times. Water was used as the dispersant medium, and the obscuration was in the range 11–16% for all measurements. The volume-based- particle size distributions, D10, D50, and D90 (where, for example, D90 is the maximum particle diameter below which 90% of the sample volume exists), were determined.

### 2.4. Preparation of Solid-Phase Products for Physical-Chemical Investigations of Products

The chosen suspensions were dried in a vacuum dryer (Binder GmbH, Tuttlingen, Germany) at 40 °C in order to gain solid products for the physico-chemical analysis.

### 2.5. Image Analysis (SEM)

The morphological features of the ground dried products were analyzed applying a scanning electron microscope (Hitachi S4700, Hitachi Scientific Ltd., Tokyo, Japan). The patterns were sputter-coated with gold–palladium under an argon atmosphere, using a gold sputter module in a high-vacuum evaporator, and the samples were investigated at 15 kV and 10 μA. The air pressure was 1.3–13 MPa. For the particle size determination of MEL, a public domain image analyzer software, ImageJ, was applied (https://imagej.nih.gov/ij/index.html, accessed on 17 November 2024) [34].

### 2.6. Structural Analysis

In order to investigate the physical state of the drug in the solid products, a Bruker D8 Advance diffractometer (Bruker AXS GmbH, Karlsruhe, Germany) system was applied with Cu K λI radiation (λ = 1.5406 Å). The samples were scanned at 40 kV and 40 mA from 3° to 40°·2θ, at a scanning speed of 0.05°/s and a step size of 0.010°. The crystallinity (xc) of MEL in the solid-state samples was ascertained semi-quantitatively in the case of both millings by the mean of the decrease in the total area beneath the curve of 3 characteristic peaks (*A_crystalline_*) compared to the rawMEL (*A_crystalline_* + *A_amorphous_*) as follows (Equation (1)):

(1) $$\mathrm{xc}=\frac{A_{crystalline}}{A_{crystalline}+A_{amorphous}}*100$$

A Mettler Toledo DSC 821e thermal analysis system with the STARe thermal analysis program V9.0 (Mettler Inc., Schwerzenbach, Switzerland) was used for the thermoanalytical investigations of samples. About 2–5 mg of pure drug or ground dried product was studied in the temperature range between 25 °C and 300 °C. The heating rate was 5 °C·min^−1^. Argon gas was used as the carrier gas at a flow rate of 10 L·h^−1^ during the DSC investigations.

### 2.7. Fourier-Transformed Infrared Spectroscopy (FT-IR)

An AVATAR330 FT-IR spectrometer (Thermo Nicolet, Unicam Hungary Ltd., Budapest, Hungary) was applied for the investigation of the formation of secondary interactions or changes in bonds in the samples. Examination was performed in the interval 400–4000 cm^−1^ at an optical resolution of 4 cm^−1^. Samples were ground and compressed into pastilles at 10 t with 0.15 g of KBr.

### 2.8. Determination of the Interparticle Interactions

Approximately 0.10 g of the samples was pressed on a 1-ton hydraulic press (Perkin Elmer hydraulic press, Specac Inc., Waltham, MA, USA). The prepared pastilles were dribbled with polar liquid (4.8 µL of purified water) and with non-polar solvent (2.0 µL of diiodomethane). A Dataphysics OCA 20 apparatus (Dataphysics Instrument GmbH, Filderstadt, Germany) was applied for the detection of the contact angle in an interval of 1 to 25 s. The contact angles of the two fluids were achieved. The surface free energy (*γ^s^*) of the products, consisting of the polar part (*γ_s_^p^*) and the disperse part (*γ_s_^d^*); (*γ^s^ = γ_s_^p^ + γ_s_^d^*) was determined based on the Wu equation [35]. The surface tension of the used liquids can be found in the literature as follows: distilled water *γ^p^* = 50.2 mN/m, *γ^d^* = 22.6 mN/m and diiodomethane *γ^p^* = 1.8 mN/m, *γ^d^* = 49 mN/m. Below, the Wu equation is shown (Equation (2)), where *θ* = contact angle, *γ* = surface free energy, *s* = solid phase, *l* = liquid phase, *d* = dispersion component, and *p* = polar component.

(2) $$\left(1+cos\theta \right)\gamma_{l}\;=\frac{4({\gamma_{s}}^{d}{\gamma_{l}}^{d})}{{\gamma_{s}}^{d+}{\gamma_{l}}^{d}}+\frac{4({\gamma_{s}}^{p}{\gamma_{l}}^{p})}{{\gamma_{s}}^{p+}{\gamma_{l}}^{p}}$$

The polarity (Pol) was calculated as the ratio of the surface free energy of the polar component and the surface free energy multiplied by 100 (Equation (3)).

(3) $$Pol=\frac{\gamma^{p}}{\gamma^{s}}*100$$

### 2.9. Characterizations of the Suspensions

Suspensions were characterized before filling into capsules.

#### 2.9.1. Resuspendability

The resuspendability of suspensions was evaluated qualitatively using a measuring cylinder of 25.0 mL. The test was performed by rotating the cylinder using a laboratory resuspending device. The number of rotations required for the complete elimination of sediment from the bottom was recorded. The suspensions were stored in a refrigerator at a temperature of 15 ± 1 °C.

#### 2.9.2. Holding Time Determination

The holding time of the samples was determined after storage for one month. The PSD of MEL in the ground suspensions was detected using laser diffraction (Malvern Mastersizer S 2000) (Malvern Instruments Ltd., Worcestershire, UK). The suspensions were stored in a refrigerator at 15 ± 1 °C. In order to resuspend the samples, a magnetic stirrer was applied. Three parallel measurements were carried out and the average values were compared with the particle sizes of the initial suspensions.

#### 2.9.3. Drug Content and Uniformity

The drug content of the suspensions was investigated by dissolving the samples containing 5.00 mg of MEL in a 25.0 mL phosphate buffer (pH 7.4 ± 0.1). The samples were stirred with a magnetic stirrer at room temperature (25 °C). After filtration (0.1 μm, FilterBio PES Syringe Filter) (Labex Ltd., Budapest, Hungary), the dissolved drug content was analyzed spectrophotometrically at 364 nm (ATI Unicam UV/VIS) (Cambridge, UK). Each suspension was sampled from 3 different locations, and the uniformity of the drug content was obtained by the calculation of the standard deviation of parallel measurements.

### 2.10. In Vitro Dissolution Test

The paddle method USP (Pharma Test, Heinburg, Germany) was applied for investigation the release of MEL from the suspensions. Dispersions containing 7.5 mg of MEL (therapeutic dose) were filled into “00” size capsules 5 sec after grinding and put quickly into the dissolution medium. The medium contained 900 mL of artificial gastric fluid, at pH 1.2 ± 0.1, and intestinal fluid (pH 6.8 ± 0.1). The rotation speed of the paddle was 100 rpm, and samples were taken at intervals up to 60 min. A spectrophotometer (ATI-UNICAM UV/VIS Spectrophotometer) was used to determine the drug content of samples at 362 nm for gastric juice and 364 nm for enteric fluid. The number of parallel runs was 3.

Dissolution efficiency (D.E.) (Equation (4)) is given by the area under a dissolution curve between defined time points and the mean dissolution time (M.D.T.) (Equation (5)) [36,37]. To facilitate the calculations, DDSolver program was applied [38].

(4) $$\mathrm{DE}=\frac{\int_{0}^{t}y*\mathrm{dt}}{y100*t}*100\%$$

(5) $$\mathrm{MDT}=\frac{\sum_{i=1}^{n}t_{i}*\Delta M_{i}}{\sum_{i=1}^{n}\Delta M_{i}}$$

In the formulas above, n is the number of sampling points; t_i_ is the ith sampling time point; y is the percentage of drug dissolved at time t; ∆M_i_ is the additional amount of drug dissolved between i and i − 1; and y100 is the maximum percentage of drug dissolved over the time period 0–t.

### 2.11. Cytotoxicity Studies

The cytotoxicity of the prepared suspensions was investigated on a Caco-2 cell line (immortalized cell line of human colorectal adenocarcinoma cells, the Caco-2 cell line was purchased from American Types Culture Collection ((ATCC) (Manassas, VA, USA)), in the range of concentration of 0.002 mg/mL–1.000 mg/mL). Cells were seeded in 96-well culture microplates (SPL Life Sciences Co., Pocheon-si, Republic of Korea) at a density of 4 × 10^4^ cells/well in DMEM (Dulbecco’s Modified Eagle’s Medium) (Sigma-Aldrich Co., St. Louis, MO, USA), supplemented with 10% FBS ((Fetal Bovine Serum) (Sigma-Aldrich Co., St. Louis, MO, USA)). The samples were incubated with the cells for 24 h at 37 °C. MTT (Sigma-Aldrich Co., St. Louis, MO, USA), dissolved in PBS (Phosphate-Buffered Saline), at a 20 μL volume was added to the wells and incubated for 4 h. Later, 10% sodium dodecyl sulfate (Reanal Laborvegyszer Kft., Budapest, Hungary), dissolved in distilled water (100 μL), was added to the wells, and the optical density (OD) was measured after a 12 h incubation at 550 nm (ref. 630 nm) with EZ REAS 400 ELISA reader (Biochrom, Cambridge, UK). Untreated cells with 100% viability served as the control. Three parallel measurements were carried out for each sample.

## 3. Results

### 3.1. Particle Size Distribution of Milled Suspensions

After the optimization of the parameters in a laboratory scale [33,39], for scaling-up, a 10-fold amount of pearls of a ground composition was used. The rotation speed was calculated based on a formula previously applied in the literature [21]. Our main purpose was to determine and compare the milling times needed for the production of nanosized MEL particles at both milling scales. First, the effect of grinding time on the particle size distribution of MEL was determined for the scale-up conditions. Based on the laser diffraction results, grinding for 70 min resulted in micronized MEL particles (D90 = 1.013 µm) (Table 2). After 90 min of milling, the particle size of the drug decreased to the submicron range (D90 = 0.697 µm) due to the high mechanical forces of the planetary ball mill and the high efficiency of the small bead size [13]. Additionally, this was aided by the PVA, which inhibits particle aggregation. Further milling did not result in a significant decrease in particle size.

**Figure 2:**
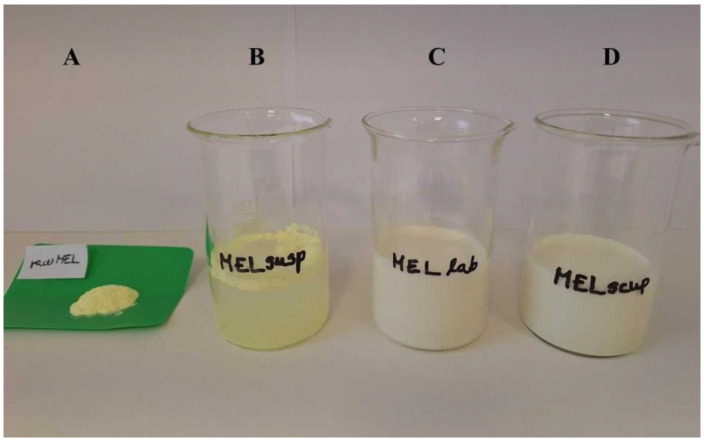
The photo of initial MEL (**A**), untreated suspended MEL (**B**), and MEL milled at different scales (**C**,**D**).

After that the effects of the lab-scale milling and the scaled-up milling on the particle size were compared (Table 3). Initial MEL, untreated suspended MEL, and MEL milled at different scales are shown in Figure 2. It can be established that the particle size of the suspended untreated MEL was lower compared with the raw drug due to the surface dissolution in the presence of PVA [40]. In these cases, the particle size was in the micrometer range. Grinding resulted in nanosized particles (D50 < 600 nm) in both cases; however, while 50 min of grinding was sufficient for nanonization in a laboratory scale, 90 min were required at the scaled-up conditions. In the case of the scaled-up milling, a 2.5-fold larger particle size was measured compared to the laboratory scale (D90 = 0.270 µm). Due to the measurement methodology of laser diffraction, particles moving together may be detected as aggreagates during size distribution determination. As a check, further investigaiotns were carried out using ImageJ software.

### 3.2. Morphology of MEL

The morphology of the ground particles is presented in the SEM images. After taking samples from the suspensions milled for 50 min (MELlab) and for 90 min (MELscup), samples were dried and characterized. The surfaces and shape changes were investigated.

**Figure 3:**
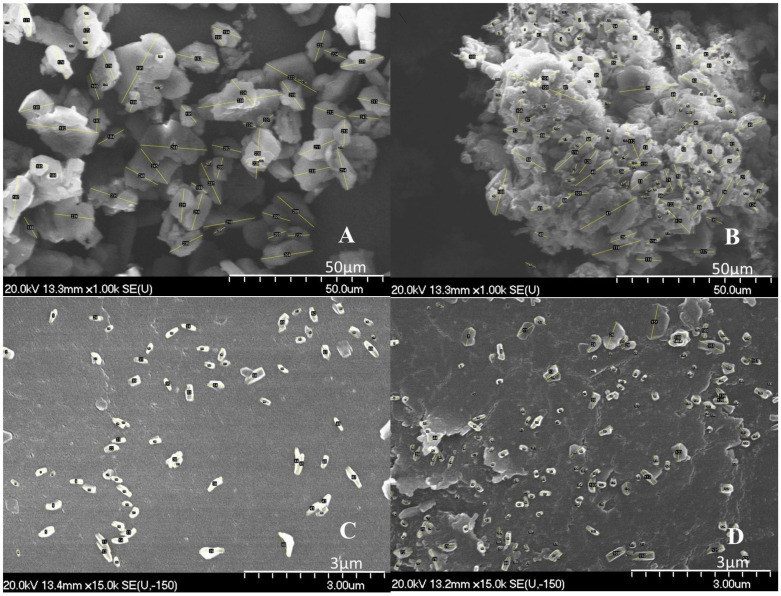
SEM pictures of rawMEL (**A**), of dried untreated suspension of MEL (**B**), of MEL milled in laboratory scale (**C**), and of MEL milled at scaled-up conditions (**D**).

RawMEL and the suspension of rawMEL contained mostly rough, lamellar crystals with a broad size distribution (Figure 3). However, ground products consisted of particles with a smooth surface and a rounded shape, as a result of the surface abrasion effect of milling beads [41]. Nanoparticles were embedded in the PVA film. Due to the presence of PVA, no aggregation was noticed in the case of the milled products. PVA coated the particles and worked to keep them away from each other [42,43].

Based on the values determined by the image analysis program ImageJ, it was established that in the dried suspension form, the particles of rawMEL (MELsusp) were separated from each other, resulting in a lower particle size in comparison with tge powder form of rawMEL, in which case the particles were stuck together (Table 4). However, in the case of dried suspensions produced by grinding (MELlab; MELscup), these values were significantly lower and more reliable (smaller standard deviation (SD)), and the average particle size of these samples was in the submicron range (<600 nm). In the SEM images, the individual particles could be analyzed separately.

### 3.3. Structural Analyses (XRPD and DSC)

**Figure 4:**
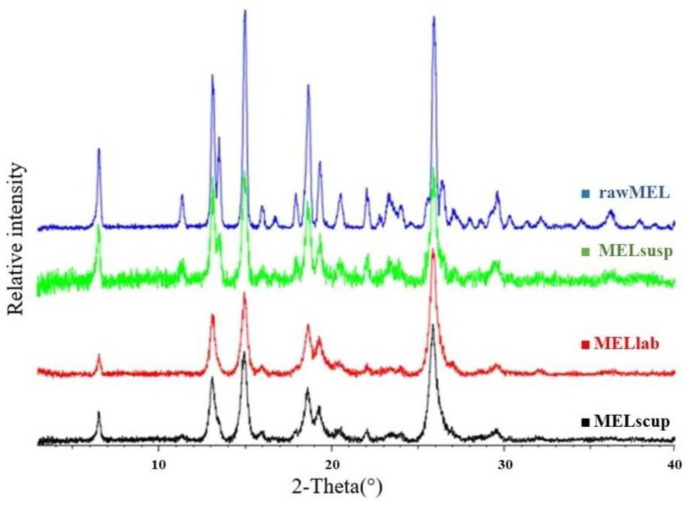
XRPD patterns of rawMEL, of dried untreated suspension of MEL, and of MEL milled at different scales.

For the characterization of the crystalline state of MEL after the grinding processes, X-ray powder diffraction was applied. RawMEL, the dried untreated suspension of MEL, and MEL milled at different scales were characterized. The XRPD diffractograms of rawMEL showed its crystalline structure. The characteristic 2θ data of MEL were 13.22, 15.06, 26.46, and 26.67. In the case of the dried untreated MEL suspension, the intensities of the characteristic peaks decreased due to the semicrystalline structure of the PVA, which covered the MEL particles [44] (Figure 4). Further grinding resulted in the same decrease in the crystallinity of the drug. After the suspendation of MEL in the PVA solution, its crystallinity decrease from 100 to 72%. This value did not change in either the laboratory scale or during scaled-up milling; the drug retained 70% of its crystalline structure (Table 5) [45].

**Figure 5:**
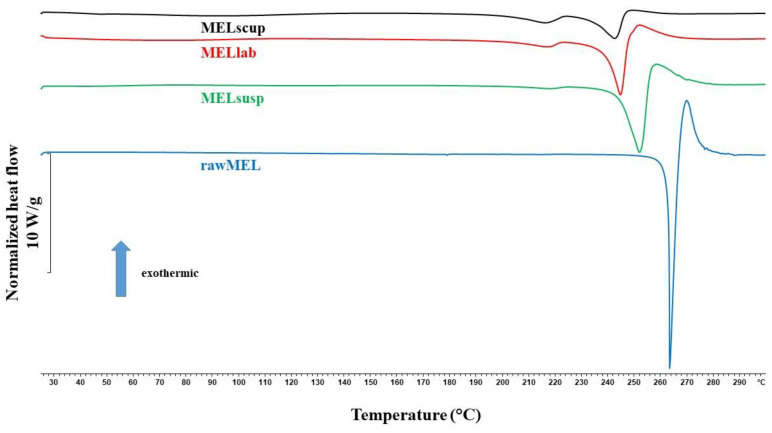
DSC thermograms of rawMEL, of dried untreated suspension of MEL, and of MEL milled at different scales.

DSC was employed to investigate the melting of MEL in the raw form, in the dried untreated suspension, and in the dried ground products (Figure 5). The DSC curve of rawMEL showed a sharp endothermic peak at 263.55 °C, representing its melting point and verifying its crystalline structure. The DSC curve revealed a broad endothermic peak for MEL in the case of the initial suspension (MELsusp: 252.23 °C) and for the milled products (MELlab: 244.94 °C and MELscup: 242.60 °C), indicating that the crystallinity of the drug was decreased. In these cases, MEL crystals melted at a lower temperature than the crystals of rawMEL. This phenomenon is due to the PVA, which covered the MEL particles during grindings and softened at 85 °C, as the glass transition temperature (Tg) value induces the melting of the drug at a lower temperature [46].

### 3.4. FT-IR Investigations

**Figure 6:**
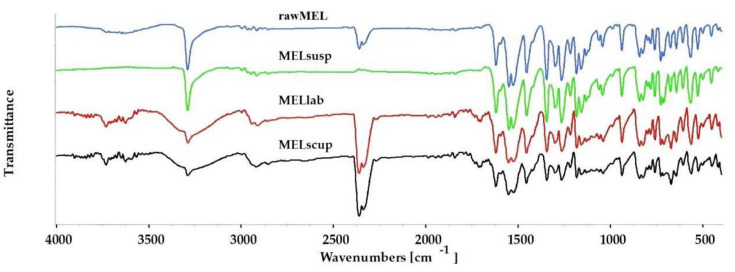
FTIR spectra of rawMEL, of dried untreated suspension of MEL, and of MEL milled at different scales.

Using FT-IR spectroscopy, we investigated whether the grinding process has any effect on the formation of secondary interactions or changes in the bonds in the samples. Figure 6 presents the FT-IR spectra of the following four samples: the rawMEL, MELsusp after drying, the ground sample (MELlab), and the scaled-up ground sample (MELscup). The most characteristic vibrations of MEL [47,48] were observed at 3288.8 cm^−1^ (N-H stretching), 1620.7 cm^−1^ (C=O stretching), 1549.8 cm^−1^ (C=N stretching), 1455.8 cm^−1^ (C=C stretching of the aromatic ring), 1344.6 cm^−1^, and 1161.6 cm^−1^ (S=O stretching vibrations of the sulfonyl groups). These characteristic peaks appeared in MELsusp as well as in the ground samples (MELlab and MELscup). There were no shifts, fusions, losses, or broadenings that would suggest the formation of new interactions, nor were there any new peaks recorded. A decrease in the intensity of the peaks was observed in samples MELlab and MELscup, which occurred due to the decrease in crystallinity and particle size [45,49]. The results of the FT-IR measurements indicated that the MEL remained in the milled preparations in an unchanged form without decomposition. The FTIR measurements proved that the milling process did not affect the molecular structure of the MEL, furthermore confirming the stability of the formulations during the milling process.

### 3.5. Interparticular Interactions

The polarity and the cohesive work (Wc) characteristics of the materials were determined on the basis of the contact angle measurements. The wettability study showed that the milled dried samples (MELlab and MELscup) had decreased values of the contact angle with water (ϴwater), indicating a more hydrophilic character as compared with hydrophobic rawMEL and MELsusp. Milling created a new hydrophobic drug surface that was hydrophilized with PVA, which is a hydrophilic polymer, and therefore, wettability could be increased [50]. The polarity of the milled products, calculated as the ratio of surface free energy of the polar component and the surface free energy, also increased, forecasting the better dissolution results (Table 6).

### 3.6. Investigations of Milled Suspnesions

#### 3.6.1. Resuspendability Test and Holding Time Determination

Resuspendability and the uniformity in particle size are essential in suspension formulation. During the resuspendability test of the investigated suspensions, it was found that both samples produced at laboratory (MELlab) and at scaled-up (MELscup) conditions proved to be stable even after 1 month of storage, as the sediment from the bottom disappeared after only two revolutions, and homogeneous suspensions were obtained. In contrast, the suspension containing untreated MEL (MELsusp) needed 45 revolutions to achieve homogeneity (Table 7). PVA increased the viscosity of the dispersion medium, thereby ensured a slow sedimentation of the nanosized particles [51].

During the holding time determination, it was obtained that no aggregation occurred after 1 month of storage (Table 8). This can be explained by the presence of PVA, which—forming the coating layer as a steric barrier around particles—could prevent aggregation and stabilize the suspension [30,52].

#### 3.6.2. Drug Content and Uniformity Determination

During the drug content investigations, a loss of more than 10% was experienced in both millings (Table 9), which can be explained by the fact that the drug may stick to the surface of the milling beads during milling, and we could not completely remove the samples from the milling chamber. The uniformity of the drug content was improved in the case of the milled samples compared to the untreated suspension.

### 3.7. Results of In Vitro Dissolution Test

**Figure 7:**
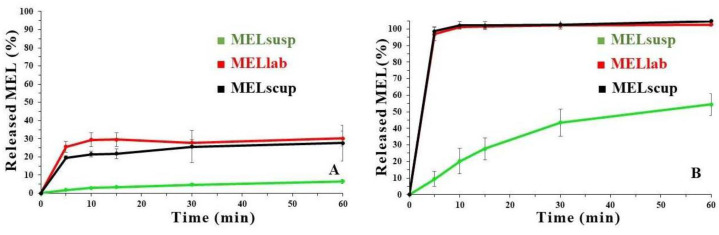
In vitro dissolution profile of initial and milled suspensions filled into capsules in artificial gastric fluid (**A**) and in artificial intestinal fluid (**B**).

First, the in vitro dissolution test was carried out in an artificial gastric fluid at pH 1.2 ± 0.1 (Figure 7A). Suspnesions of MEL were filled into the capsules and promptly put into the medium. MELsusp was compared with the ground products. The investigation exhibited the slow and gentle dissolution of the drug from the untreated MELsusp, and after 60 min, approximately 7% of the drug was liberated. MEL has a weak acidic character (pKa 4.08), which results in a very low solubility in gastric fluid (1.6 ± 0.2 mg/L, at 37 °C) [53]. Grinding in both laboratory and scaled-up cases resulted in a 10-fold increase in the dissolution rate of the drug. In the case of the MELlab sample, 20% of the drug was dissolved in the first 10 min. In case of MELscup, this value was 10%; however, after 30 min, 20% of MEL was dissolved in this case too. This increase in the dissolved amount resulted in the small particle size and the wetting effect of PVA, which improves the wettability of poorly soluble drugs, enhancing their dispersion in aqueous environments [51].

After the dissolution test in the artificial gastric fluid, dissolution investigations were carried out in an artificial intestinal fluid (pH 6.8 ± 0.1) (Figure 7B). In this case, the liberated amount of the drug was higher because of the higher solubility of MEL (0.272 ± 0.001 mg/mL, at 37 °C). It was established that in case of MELsusp, the rate of dissolution of raw MEL, with particles in the micrometer size range, was lower compared with ground products; only 10% of the drug was dissolved in the first 5 min and approximately 50% after 60 min. Milled products had a significantly better drug release than the reference sample. Furthermore, it can be observed that there was no significant difference between the liberated drug amount from the samples prepared in laboratory scale and at scaled-up conditions. About 100% of the drug released from both ground samples in the first 5 min due to the small particle size and the enhanced wettability increased with the use of PVA [54].

During the determination of dissolution kinetics, the dissolution efficiency (DE) and the mean dissolution time (MDT) were determined. The calculated values in the table clearly show that the dissolution of MEL and the average dissolution time in the case of ground samples are coincident for intestinal fluid and significantly better than those of the reference for both digestive media. More than 50% lower values were calculated for MDT in the case of the intestinal fluid compared with gastric fluid (Table 10).

### 3.8. Cytotoxicity Studies

**Figure 8:**
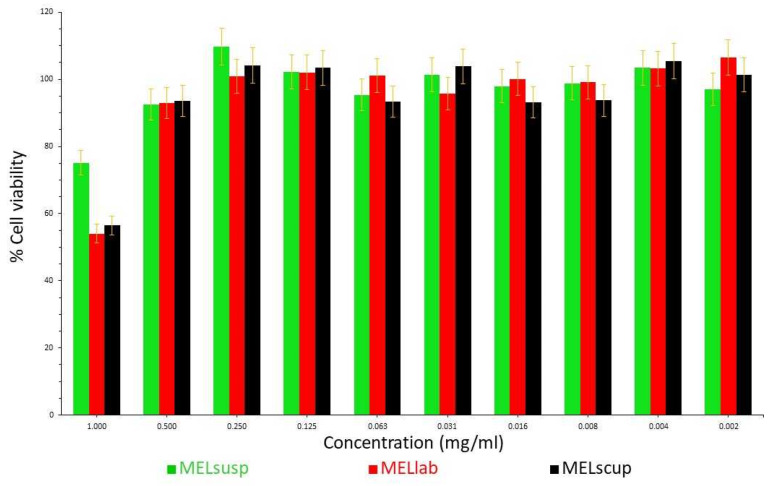
Cytotoxicity of the untreated and milled suspensions as a function of concentration on a Caco-2 cell line.

The therapeutic dose of MEL in marketed medicines is 7.5 or 15.0 mg per os [55]; hence, in this study, suspensions containing 7.5 mg of MEL were chosen for further investigations. In order to recognize the effect of nanoparticles on cells, cytotoxicity analyses were performed. The cytotoxicity of the prepared suspensions (MELsusp, MELlab and MELscup) was determined on a Caco-2 cell line (Figure 8). It was observed that the degree of cell death appreciably increased above 0.5 mg/mL MEL concentration, especially in case of samples prepared by grinding. However, below concentration values of 0.5 mg/mL, it can already be stated that the suspensions can be used safely; there is no need to expect a significant toxic effect. Our capsules contained the drug in a concentration of 0.1 mg/mL; therefore, no cytotoxic effect was expected in the case of the investigated samples.

## 4. Conclusions

In our work, the possibility of a scaling-up of a combined wet grinding technique—previously optimized by our research group—was investigated. MEL was used as a NSAID. MEL-containing samples were prepared at laboratory and at tenfold scaled-up conditions. Water was used as a grinding medium; therefore, the procedure was considered green technology, while the product was organic solvent-free. The effect of millings on the physico-chemical properties of the products were determined. The resuspendability, holding time, drug content, and uniformity of suspensions were established. The results of the dissolution tests were compared. For both lab-scale milling and scaled-up milling, the particle size of the suspensions was in the submicron range. For nanonization, 50 min of milling at laboratory conditions, and 90 min of milling at scaled-up conditions were needed. Both grindings resulted in rounded particles with a smooth surface. The maximum particle size determined by ImageJ analyses program was almost the same (MELlab = 0.57 µm; MELscup = 0.58 µm) in both cases. A 30% decrease in the drug crystallinity was confirmed by DSC and XRPD during the grinding procedures. No secondary interaction could be detected between the compounds in the investigated products for any milling. The polarity of the milled products increased compared with the untreated drug. Formulation investigations proved the physical stability of ground suspensions. In the intestinal fluid, the dissolved amount of MEL was the same for any sampling points under laboratory conditions and under scaled-up conditions too. The liberated drug amount was significantly higher compared with the results of the gastric fluid. The samples investigated were not found to be cytotoxic.

We can declare based on our results that, although there is no suggested protocol for scaling-up the grinding process and a lot of experiments are needed even under laboratory conditions, the settings of the grinding speed and the grinding time are essential during the scale-up procedure. Furthermore, the application of hydrophilic additives is usually required to ensure the desired particle size reduction. Despite the size limitation of our planetary ball mill compared to industrial-scale grinding equipment, this report provides useful information and can be a starting point for the industry, where the scale-up of different processes—including grinding—is essential to attain cost-effective production.

## Figures and Tables

**Table 1:** Abbreviations of samples in the text.

rawMEL	initial drug
MELsusp	untreated suspension of MEL
MELlab	sample milled in a laboratory scale for 50 min at 500 rpm
MELscup	scaled-up sample (335 rpm, 90 min)

**Table 2:** PSD of MEL in suspensions prepared by scale-up method.

Samples	D10 (µm)	D50 (µm)	D90 (µm)
rawMEL	11.40	34.26	73.59
10 min	2.115	3.112	3.690
30 min	0.150	0.204	0.255
50 min	1.101	1.218	1.329
70 min	0.810	0.905	1.013
90 min	0.517	0.590	0.697
110 min	0.518	0.594	0.696
130 min	0.517	0.589	0.696
150 min	0.517	0.589	0.695

**Table 3:** PSD of initial MEL, of untreated suspended MEL, and of MEL milled at different scales.

Samples	D10 (µm)	D50 (µm)	D90 (µm)
rawMEL	11.40	34.26	73.59
MELsusp	11.01	20.407	35.28
MELlab	0.093	0.177	0.270
MELscup	0.517	0.590	0.697

**Table 4:** PS data of rawMEL, of dried untreated suspension of MEL, and of MEL milled at different scales determined by ImageJ.

Samples	Minimum (µm)	Maximum (µm)	Average (µm)
rawMEL	3.19 ± 1.32	27.05 ± 8.15	12.01 ± 6.07
MELsusp	0.60 ± 0.09	16.06 ± 6.36	4.62 ± 3.05
MELlab	0.14 ± 0.01	0.57 ± 0.02	0.33 ± 0.01
MELscup	0.09 ± 0.02	0.58 ± 0.05	0.24 ± 0.03

**Table 5:** The degree of MEL crystallinity (%) before and after milling procedures.

Sample	Crystallinity (%)
rawMEL	100
MELsusp	71.8
MELlab	70.2
MELscup	71.2

**Table 6:** The polarity, the surface free energy (γ), and the contact angle values of the rawMEL, dried untreated, and dried milled suspensions.

Samples	θ Water (°)	θ Diiodomethane (°)	γ (mN/m)	Polarity (%)
rawMEL	68.99	13.33	57.10	20.96
MELsusp	67.47	9.46	58.29	21.56
MELlab	61.97	27.02	58.76	26.67
MELscup	54.40	21.03	62.40	31.07

**Table 7:** Resuspendability of the untreated and milled suspensions.

Sample	Number of Revolutions
MELsusp	45
MELlab	2
MELscup	2

**Table 8:** Average particle size of milled suspensions after 1 month of storage.

Sample	Average Particle Size (nm)	
	Day 0	Day 30
MELlab	296.1 ± 20.3	299.3 ± 17.1
MELscup	424.7 ± 25.6	428.6 ± 45.2

**Table 9:** Drug content and uniformity of the untreated and milled suspensions.

Sample	Drug Content and Uniformity (mg)	Drug Content (%)
MELsusp	5.00 ± 0.35	100 ± 7.07
MELlab	4.41 ± 0.06	88.12 ± 1.36
MELscup	4.44 ± 0.03	88.80 ± 0.63

**Table 10:** Dissolution kinetics of the untreated and milled suspensions.

	Gastric Fluid		Intestinal Fluid	
Samples	DE (%)	MDT (min)	DE (%)	MDT (min)
MELsusp	4.37	20.05	37.03	19.17
MELlab	27.48	6.96	97.37	3.14
MELscup	23.57	8.70	98.48	3.63

## Data Availability

Data are contained within the article.
